# Letrozole versus laparoscopic ovarian drilling in clomiphene citrate-resistant women with polycystic ovary syndrome: a systematic review and meta-analysis of randomized controlled trials

**DOI:** 10.1186/s12958-019-0461-3

**Published:** 2019-02-06

**Authors:** Qiong Yu, Shifu Hu, Yingying Wang, Guiping Cheng, Wei Xia, Changhong Zhu

**Affiliations:** 1 Reproductive Medicine Center, Tongji Hospital, Tongji Medical College, Huazhong University of Science and Technology, Wuhan, 430030 Hubei China; 2 Family Planning Research Institute, Tongji Medical College, Huazhong University of Science and Technology, Hangkong Road 13, Wuhan, 430030 Hubei China; 3 Reproductive Medicine Center, Tongji Medical College, Huazhong University of Science and Technology, Wuhan, 430030 Hubei China

**Keywords:** Letrozole, Laparoscopic ovarian drilling, Polycystic ovary syndrome, Ovulation induction

## Abstract

The objective of this systematic review was to examine the literature and to compare the effectiveness of letrozole (LE) versus laparoscopic ovarian drilling (LOD) for the induction of ovulation in women with clomiphene citrate (CC)-resistant polycystic ovary syndrome (PCOS). The PUBMED, Web of Science, and EMBASE databases were searched systematically for eligible randomized controlled trials (RCTs) from English language articles published from database inception to September 2018. Data were independently extracted and analyzed using the fixed-effects model or random-effects model according to the heterogeneity of the data. Four RCTs including 621 patients (309 in the LE group and 312 in the LOD group) met the inclusion criteria. There were no differences with regard to ovulation rate (relative risk [RR] 1.12; 95% confidence interval [CI] 0.93 to 1.34; *P* = 0.12, I^2^ = 90%, 541 patients, three studies), pregnancy rate (RR 1.21; 95% CI 0.95 to 1.53; *P* = 0.12, I^2^ = 0%, 621 patients, four studies), live birth rate (RR 1.27; 95% CI 0.96 to 1.68; *P* = 0.09, I^2^ = 19%, 541 patients, three studies), and abortion rate (RR 0.7; 95% CI 0.3 to 1.61; *P* = 0.40, I^2^ = 0%, 621 patients, four studies) between the two groups. These results indicated that LE and LOD appear to be equally effective in achieving live birth rate in patients with CC-resistant PCOS.

## Introduction

Polycystic ovary syndrome (PCOS) is one of the most common endocrine pathologies and is a frequent cause of anovulatory infertility affecting 5 to 8% of reproductive age women [1, 2]. The main syndromes of PCOS are chronic anovulation, hyperandrogenism, obesity, hypertension, diabetes mellitus type 2, and insulin resistance [3–5]. Induction of ovulation is considered an essential treatment option for PCOS, and clomiphene citrate (CC) remains the first-line drug for the induction of ovulation among infertile women with PCOS. However, 15–40% of women who do not respond to increasing doses of CC and fail to ovulate are defined as being CC resistant [6]. Currently, The potential reproductive benefits of metformin, a drug endowed with the capacity to ameliorate insulin resistance in polycystic ovary syndrome (PCOS), has garnered much interest over the past 2 decades [7]. Furthermore, the most frequently administered therapeutic treatments in patients with CC-resistant PCOS include gonadotropin, laparoscopic ovarian drilling (LOD), and aromatase inhibitors [8–10].

Letrozole (LE), an orally active, reversible, nonsteroidal aromatase inhibitor, has good potential for inducing ovulation in women with PCOS without exerting antiestrogenic effects on the endometrium [11]. Additionally, LE has a short half-life (45 h) and thus is rapidly eliminated from the body [12]. Furthermore, LE is effective for the induction of ovulation in women with PCOS who do not conceive with CC [9, 13, 14]. LOD is currently accepted as a successful second-line treatment for the induction of ovulation in CC-resistant PCOS [15, 16]. Recently, the efficacy of LE compared with LOD treatment as therapy for CC-resistant PCOS patients has been studied in randomized controlled trials (RCTs). However, the conclusions have not been completely consistent. For example, previous studies revealed that LE was superior to LOD in regard to the ovulation rate [14, 17], but other studies revealed that LE and LOD were equally effective in inducing ovulation and achieving pregnancy among patients with CC-resistant PCOS [18, 19]. The recent systematic review comparing LE with LOD for subfertile women with CC-resistant PCOS indicated that there were no differences with regard to live birth rates, pregnancy rates and miscarriage rate [20]. However, the clinical utility of this meta-analysis is uncertain due to the treatments in the included RCTs were different, which may lead to an increased risk of bias.

The purpose of the present meta-analysis was to systematically examine the literature and identify the results of RCTs to provide evidence for the effectiveness of LE compared with LOD treatment for the induction of ovulation in CC-resistant PCOS patients.

## Materials and methods

### Search strategy

The literature search of this systematic review was conducted independently by Qiong Yu, Shifu Hu, and Yingying Wang on major electronic databases including PubMed, Web of Science, and EMBASE from the earliest record in each database to September 2018. The key words used were “polycystic ovary syndrome,” “PCOS,” “PCO,” “letrozole,” “LE,” “aromatase inhibitors,” “clomiphene citrate,” “laparoscopic ovarian drilling,” “LOD,” “randomized controlled trial,” and “RCT.” The search was restricted to articles published in English. Additionally, hand screening of the references of the included articles was carried out to identify additional studies.

### Inclusion and exclusion criteria

Articles were considered eligible if they met the following criteria: (1) an RCT design; (2) PCOS diagnosed as fulfilling the Rotterdam 2003 criteria [21, 22]; (3) studies on clinical trials in humans; (4) CC resistance; (5) an intervention of LE versus LOD for the induction of ovulation in women with PCOS; and (6) reporting at least one of the following outcomes: ovulation rate (calculated as the number of ovulatory cycles divided by the total number of menstrual cycles), pregnancy rate (calculated as the number of clinical pregnancies divided by the number of patients), endometrial thickness at human chorionic gonadotrophin (HCG) injection, live birth rate (calculated as the number of live births divided by the number of patients), and miscarriage rate (calculated as the number of first-trimester spontaneous abortions divided by the number of patients). The exclusion criteria were (1) case reports, review articles, expert opinions, letters, or observational studies; (2) non-RCTs; (3) patients without a diagnosis of PCOS or infertility of unknown cause; and (4) non-CC resistance.

### Data extraction and quality assessment

Data were extracted independently from the included studies by two investigators (Shifu Hu and Guiping Cheng) and recorded in a spreadsheet as follows: first author name(s), publication year, country, intervention procedures, number of cases, and main outcome parameters. We used the Cochrane Collaboration tool to evaluate the quality of all of the selected studies. Studies with good-quality criteria addressed the following elements: random sequence generation, allocation concealment, blinding, incomplete outcome data, selective reporting, and other bias. Discrepancies were resolved through consultation and discussion with a senior reviewer (Wei Xia or Changhong Zhu) when necessary.

### Statistics and data analysis

RevMan ver. 5.2 was used to perform the statistical analysis. Statistical heterogeneity was evaluated according to the values of *P* and *I*^2^ using the standard chi-square Q test. If *I*^2^ < 50% and *P* < 0.05, which indicated low or moderate heterogeneity, a fixed-effects model was calculated with use of the Mantel-Haenszel test for meta-analysis. Otherwise, a random-effects model was applied. Dichotomous outcomes were calculated using the relative risk (RR) with 95% confidence interval (CI), and continuous outcomes were calculated using the standardized mean difference (SMD) with 95% CI. Funnel plots were used to evaluate publication bias. A *P* value of less than 0.05 was considered to be statistically significant.

## Results

### Study characteristics and quality assessment

**Fig. 1:**
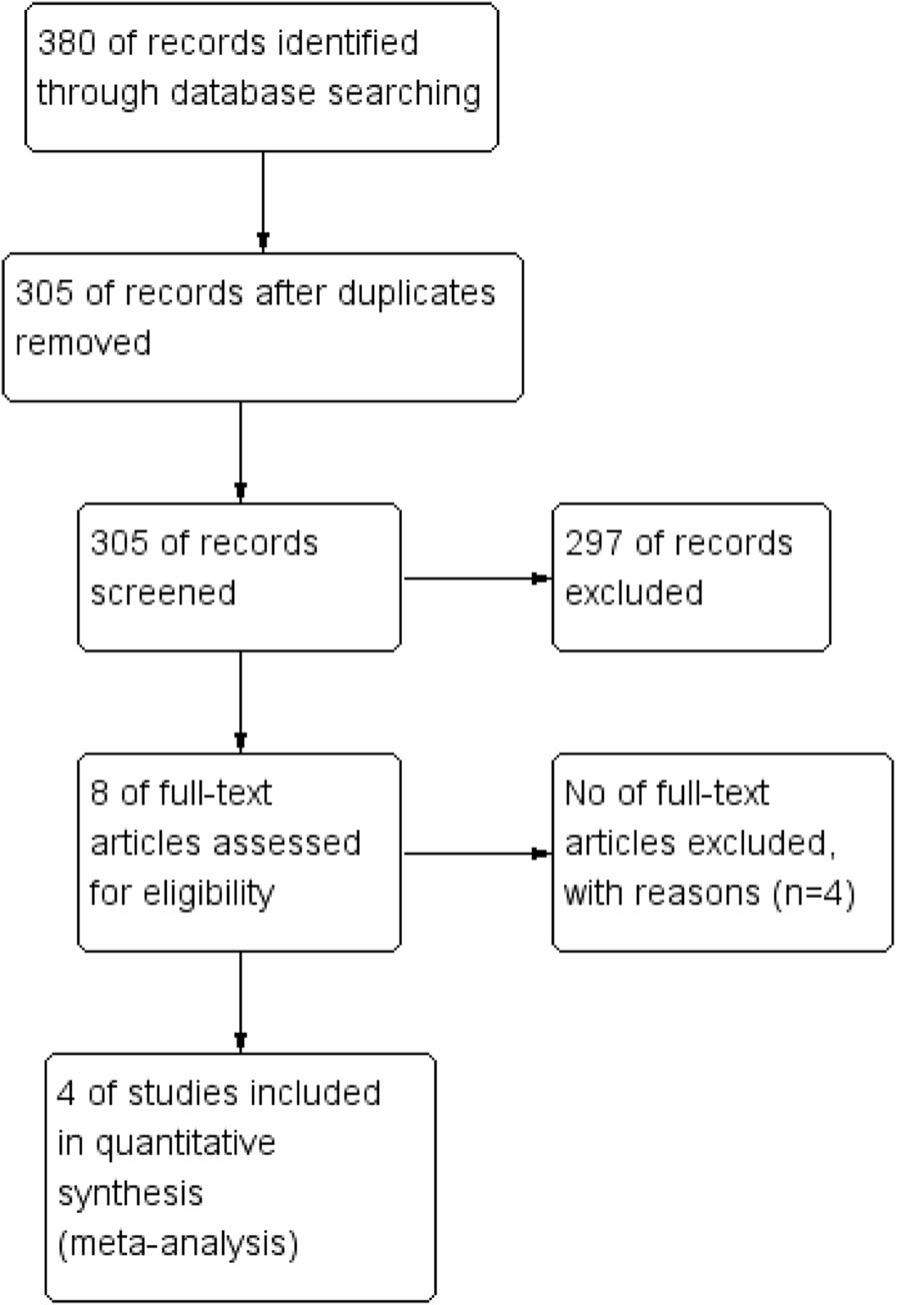
Flowchart of study selection of the randomized controlled trials (RCTs)

The literature selection process is presented in Fig. 1. In total, 380 articles were identified in the initial database search. After screening of abstracts and titles, 372 apparently irrelevant articles were excluded because they did not meet the inclusion and/or exclusion criteria. In addition, four articles were excluded after full text review as shown in Fig. 1. Eventually, four eligible studies [14, 17–19] including 621 patients were considered for this meta-analysis. Of these patients, 309 and 312 were classified into the LE and LOD groups, respectively. The process of randomization was sufficient in all studies [14, 17–19]. Allocation sequence concealment was implemented and reported in only two studies [14, 19] and was unclear in the remainder [17, 18]. The participants and researchers were distinctly masked to the intervention in only one study [19], however, most of the remaining cases masking was unclear in the remaining three studies [14, 17, 18]. Both the characteristics of the studies included in the meta-analysis and the quality assessment of the included RCTs are presented in the supplemental material (Tables 1 and 2, respectively).

**Table 1:** Characteristics of the studies included in the review

Author (year)	Country	Interventions	Patients (n)	Cycles (n)	Outcomes included in the meta-analysis
Abu Hashim (2010)	Egypt	2.5 mg LE LOD	128 132	512 525	Ovulation rate, miscarriage rate, pregnancy rate, live birth rates
Liu (2015)	China	2.5 mg LE LOD	71 70	382 358	Ovulation rate, endometrial thickness, abortion rate, pregnancy rate, live birth rate
Abdellah (2011)	Egypt	5 mg LE LOD	70 70	346 373	Endometrial thickness, ovulation rate, miscarriage rate, pregnancy rate, live birth rate
Ibrahim (2017)	Egypt	2.5 mg LE LOD	40 40	40 40	Ovulation rate, abortion rate, pregnancy rate

*LE* letrozole, *LOD* laparoscopic ovarian drilling

**Table 2:** Quality assessment of the included studies

Author (year)	Random sequence generation	Allocation concealment	Blinding of participants and personnel	Blinding of outcome assessment	Incomplete outcome data	Selective reporting	Other bias
Abu Hashim (2010)	Yes	Yes	Yes	Yes	Yes	No	Yes
Liu (2015)	Yes	No	No	No	No	No	Yes
Ibrahim (2017)	Yes	No	No	No	No	Yes	Yes
Abdellah (2011)	Yes	Yes	No	No	No	No	Yes

### Ovulation rate

**Fig. 2:**
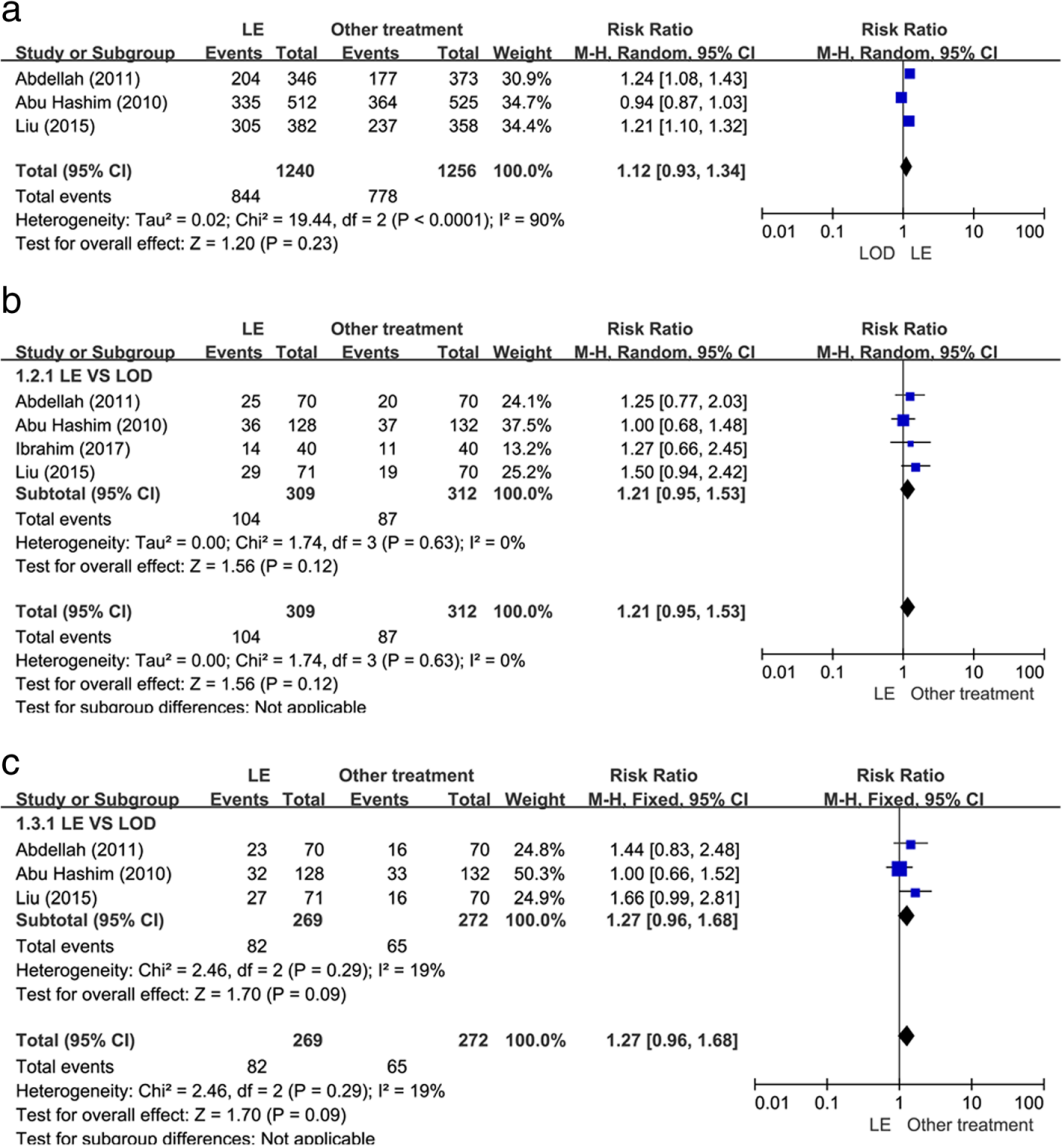
Letrozole (LE) versus laparoscopic ovarian drilling (LOD): rates of ovulation and pregnancy. (**a**) Ovulation rate. (**b**) Pregnancy rate. (**c**) Live birth rate

Three studies [14, 17, 19] evaluated the ovulation rate. As shown in Fig. 2a, there was no statistically significant difference between the LE group and LOD group when comparing ovulation rates (RR 1.12; 95% CI 0.93 to 1.34; *P* = 0.12, I^2^ = 90%, 541 patients).

### Pregnancy rate

As presented in Fig. 2b, all four of the studies [14, 17–19] including 621 patients reported data on pregnancy rate. There was a statistically significant increase in the pregnancy rate in the LE group when compared with the LOD group (RR 1.21; 95% CI 0.95 to 1.53; *P* = 0.12, I^2^ = 0%).

### Live birth rate

The three studies [14, 17, 19] that evaluated live birth rate included 541 patients. As shown in Fig. 2c, there was no statistically significant difference when comparing LE with LOD in these studies (RR 1.27; 95% CI 0.96 to 1.68; *P* = 0.09, I^2^ = 19%).

### Abortion rate

**Fig. 3:**
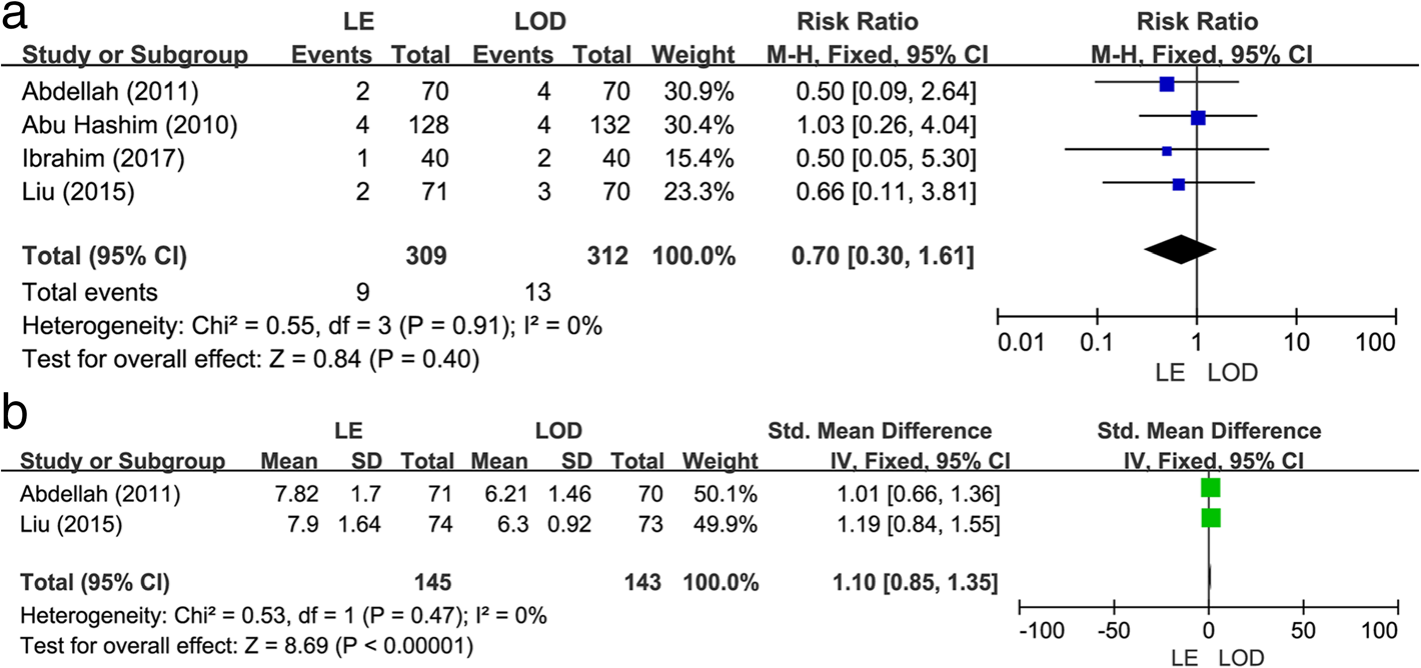
Letrozole (LE) versus laparoscopic ovarian drilling (LOD): abortion rate and endometrial thickness at human chorionic gonadotrophin (HCG) injection. (**a**) Abortion rate. (**b**) Endometrial thickness at HCG injection

As shown in Fig. 3a, four studies [14, 17–19] including 621 patients reported on abortion rate. There was no significant difference in the abortion rate between LE versus LOD treatment in the studies (RR 0.7; 95% CI 0.3 to 1.61; *P* = 0.40, I^2^ = 0%).

### Endometrial thickness

Two studies [14, 17] reported the endometrial thickness on the day of HCG injection. As shown in Fig. 3b, there was no obvious heterogeneity across the studies (I^2^ = 0%, *P* = 0.48), and thus a fixed-effects model was used. The meta-analysis revealed a statistically significant increase in the endometrial thickness in the LE group relative to the LOD group (SMD 1.10; 95% CI 0.85 to 1.35; *P* < 0.00001, Fig. 3b).

### Publication bias

**Fig. 4:**
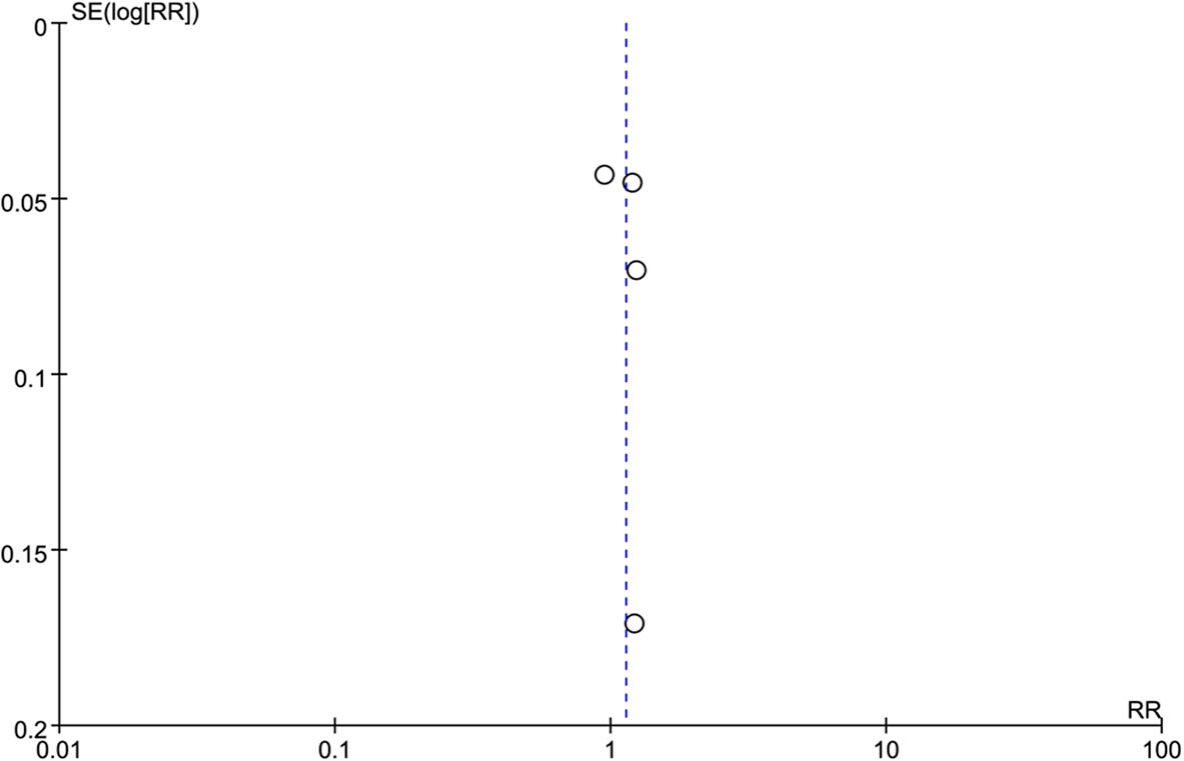
Funnel plot for detecting publication bias of all four included studies

A funnel plot was generated to qualitatively evaluate publication bias. The funnel plot for the outcome pregnancy rate shown in Fig. 4 is almost symmetrical, indicating that there was no potential publication bias in the four included studies.

## Discussion

Drug-induced ovulation is consistently proposed for infertile women with anovulatory PCOS, and CC-resistant PCOS is a challenge to treat. Currently, the traditional options for the induction of ovulation in infertile women with CC-resistant PCOS comprise gonadotropins and LOD [8]. However, researchers found that CC-resistant PCOS patients may instead benefit from LE [14, 17]. Abdellah [14] showed that LE significantly increase the likelihood of ovulation when compared with LOD in CC-resistant PCOS patients (RR 1.24, 95% CI 1.08 to 1.43). This finding was in accordance with another RCT recently published by Liu et al. [17].

In the present meta-analysis, we evaluated the effectiveness of LE versus LOD treatment to improve fertility outcomes in women with CC-resistant PCOS on the basis of the available RCTs. Our data synthesis revealed that there was insufficient evidence to establish a difference between LE and LOD in terms of ovulation, pregnancy, and rates of live births and abortion in patients with CC-resistant PCOS. Furthermore, the present meta-analysis included two studies showing that a greater endometrial thickness was detected in the LE group than in the LOD group. These results are in agreement with previous studies [23, 24]. Patients taking LE probably have a thicker endometrium because they lack the anti-estrogenic effects occurring in the follicular phase and because of the shorter life span of LE [25]. However, there is no correlation between endometrial thickness and efficacy [26, 27].

In addition to evaluating the clinical effects, cost should be considered to make a rational treatment decision. Medical cost may be a determining factor in the choice of a treatment for the induction of ovulation in CC-resistant PCOS patients. Economic analyses (including cost-utility, cost-effectiveness, and cost-benefit analyses) of these strategies have been rarely determined for the induction of ovulation owing to the lack of published evidence of economic outcomes. Of the studies included in our analysis, only Abu Hashim et al. [19] reported that the cost of ovulation induction per cycle for LE and LOD was 185 versus 1500 Egyptian pounds, respectively, indicating that LE would appear to be financially preferable. Future studies are needed to elucidate and compare the economic outcomes of different treatments for the induction of ovulation to help clinicians decide whether to use LE for individual patients. Only then can a meaningful conclusion be made as to the economic benefits and subsequent impact on individual healthcare.

Clinically, the comparative efficacy of LE and LOD is significant. LOD is currently accepted as a successful second-line treatment for the induction of ovulation in CC-resistant PCOS [28–30]. However, LOD requires surgical induction of ovulation, hospital treatment, and general anesthesia and may lead to the risks of pelvic adhesions and a decrease in ovarian function, and hinder any subsequent pregnancies [14, 31]. LE, a highly selective and competitive aromatase inhibitor, has been found to be an effective, and well-established therapy for infertility in CC-resistant PCOS [9, 13]. However, the safety of LE has raised a heated discussion and due to lack of RCTs assessing safety issues. Previous data by Biljan et al. [32] indicated an increased risk of congenital anomalies in letrozole treated babies, nevertheless recent data from prospective and retrospective trials [33, 34] have opposed these initial findings and supported the safety of LE comparing with traditional ovulation induction treatment. Furthermore, the cost of treatment is another problem. Preliminary data by Abu Hashim [19] indicated that the cost of letrozole per cycle is much lower compared with the hospital charges needed for LOD (185 vs. 1500 Egyptian pounds, respectively). The results of the current meta-analyses indicated that LE is as equally effective as LOD for infertility in CC-resistant PCOS.

The strength of the present study is that it provides quantitative evaluations of the efficiency of LE versus LOD treatment in the induction of ovulation in CC-resistance PCOS patients. The methodology used in this meta-analysis rigorously followed the Cochrane guidelines, and all eligible studies were prospective RCTs. The funnel plot did not show any publication bias, which indicates a good research strategy. Furthermore, the baseline characteristics of the participants in the RCTs were basically comparable, indicating a representative patient population. Finally, we followed the Rotterdam 2003 criteria to formulate the inclusion criteria and extract data from all four of the included studies. Therefore, we are convinced that the chance of reviewer mistakes and the introduction of reviewer bias have been minimized.

Several limitations to this meta-analysis should, however, be mentioned. First, there were only four eligible RCTs, and some of the included studies had a small number of participants, which may reduce the reliability and validity of the conclusions. Second, the literature search was restricted to studies published in the English language, which might have introduced a language bias. Third, the dosage of LE was not exactly the same in the four studies analyzed, so the quality of the evidence was relatively low for the summarized estimates. Four, although all of the included studies were RCTs, some of them did not elucidate the randomization, blinding allocation, and concealment methods, which might have resulted in high risk of publication and reporting bias. Therefore, further evidence from high-quality adequately powered RCTs with larger sample sizes are necessary to assess which kind of treatment (LE or LOD) is the most efficacious in inducing ovulation in CC-resistant PCOS patients.

## Conclusion

Despite the aforementioned limitations, the present results of this systematic meta-analysis showed that LE treatment is equally as effective as LOD in treating infertility in CC-resistant PCOS patients.
